# Decision-making after continuous wins or losses in a randomized guessing task: implications for how the prior selection results affect subsequent decision-making

**DOI:** 10.1186/1744-9081-10-11

**Published:** 2014-04-03

**Authors:** Guangheng Dong, Xiao Lin, Hongli Zhou, Xiaoxia Du

**Affiliations:** 1 Department of Psychology, Zhejiang Normal University, 688 Yingbin Road, Jinhua, Zhejiang, P.R. China; 2 Department of Physics, Shanghai Key Laboratory of Magnetic Resonance, East China Normal University, Shanghai, P.R. China

## Abstract

**Background:**

Human decision-making is often affected by prior selections and their outcomes, even in situations where decisions are independent and outcomes are unpredictable.

**Methods:**

In this study, we created a task that simulated real-life non-strategic gambling to examine the effect of prior outcomes on subsequent decisions in a group of male college students.

**Results:**

Behavioral performance showed that participants needed more time to react after continuous losses (LOSS) than continuous wins (WIN) and discontinuous outcomes (CONTROL). In addition, participants were more likely to repeat their selections in both WIN and LOSS conditions. Functional MRI data revealed that decisions in WINs were associated with increased activation in the mesolimbic pathway, but decreased activation in the inferior frontal gyrus relative to LOSS. Increased prefrontal cortical activation was observed during LOSS relative to WIN and CONTROL conditions.

**Conclusion:**

Taken together, the behavioral and neuroimaging findings suggest that participants tended to repeat previous selections during LOSS trials, a pattern resembling the gambler’s fallacy. However, during WIN trials, participants tended to follow their previous lucky decisions, like the ‘hot hand’ fallacy.

## Introduction

Decision-making is a goal-directed cognitive function involving the processes to choose from available options associated with varying levels of risk, uncertainty and reward [1]. Decision-making under risk conditions is important for adaptive behaviors [2]. However, the decision-making process is often affected by prior selections and their outcomes [3-6], even when subjects know that trials are independent and outcomes are random [7,8]. A recent study showed that participants were more risk seeking after losing a gamble than after winning a gamble [3]. This pattern of increased risk-taking following losses has been proposed to arise from a cognitive distortion termed the ‘gambler’s fallacy’, which is the belief that if deviations from expected behaviors are observed in repeated independent trials of some random process, future deviations in the opposite direction are then more likely.

Risky decision-making refers to choices that may result in reward or punishment yet the outcome is not fully predictable. Laboratory tasks often model risk-taking in two ways. One way is that the risk level (i.e. probability of a reward versus punishment) varies while the amount of potential reward or punishment remains stable. Xue’s study investigated the risk taking level after varying levels of wins or losses, and reported gambler’s fallacy during decision-making process [3]. Another way is that the risk level remains stable while the reward/punishment outcomes are random and trials are independent, as with coin tosses where the probability of a win or loss is always 50% for each independent trial [9]. The aim of the current study was to assess whether previous outcomes (continuous wins or losses) would affect subsequent decision-making and its neural correlates as assessed using functional magnetic resonance imaging (fMRI) while the risk level is held constant.

Psychological studies on the gambler’s fallacy have primarily viewed it as a cognitive bias produced by a psychological heuristic: people believe short sequences of random events should be representative of longer ones [10]. In this study, we designed decision-making task for fMRI. This task modeled continuous wins or losses using a simple gambling paradigm, and would allow us to assess how prior outcomes (gain vs. loss) affecting subsequent decision-making. Although participants were informed that the outcomes of their choice during the task were ‘random’, they were actually organized into series of continuous wins (WIN), continuous losses (LOSS), or pseudo-random non-continuous outcomes (CONTROL). The WIN and LOSS series were included to create the effect from previous outcomes.

As a consequence of experimental confounds that are related to decision processes, some brain regions, such as the inferior frontal gyrus/anterior insula (IFG/AI) and anterior cingulate cortex (ACC), are involved in risky decision making [11-14]. Thus, the brain features in the IFG and ACC are on the focus of our study. At least three cognitive processes are hypothesized to be involved during performance of such a decision-making task: decision process (reward-seeking/punishment-avoidance), executive inhibition, and processing of reward/punishment (feedback). Recent neuroimaging studies have identified the neural activities of decision-making under risk [15-17]. First, in healthy subjects, increased reward exposure is associated with increased brain activity in mesolimbic regions [18,19], which has been demonstrated to be sensitive to reward and punishment [20,21]. Second, the prefrontal cortex is implicated in inhibition and contextual analysis in resolving the uncertainty, while the striatum plays a role in the evaluation of prospective risky outcomes [22,23]. Third, the caudate has been demonstrated to be highly involved in learning and memory, particularly regarding feedback processing [24]. The activity in caudate can be expected in our study because the continuous outcomes will bring strong feedbacks.

The present task allowed us to test three independent hypotheses. First, continuous wins or losses would heighten participants’ desire to win in the subsequent trial. We hypothesized that continuous wins would reinforce their gambling behaviors (which could be indexed by changes in response time), and would be accompanied by increased activities in mesolimbic regions. Second, continuous losses would elicit frustration that would affect subsequent decision-making, and which would associate with a greater activation in the frontal regions. Third, the continuous wins would bring participants the fallacious belief of the success in the coming attempts, as known the ‘hot hand fallacy’ [25], which is the belief that one success with a random event indicates a good chance of further success in additional attempts. Therefore, we hypothesized that participant’s feedback related brain regions, such as caudate [26,27] might show higher activation after continuous wins.

## Methods

### Participant selection

The Human Investigations Committee at Zhejiang Normal University approved this study (zjnuhe09062). All participants provided written informed consent. Thirty-one male subjects (22.8 ± 3.4 years) participated in this study. This study focused on male participants because of the higher prevalence rates of gambling in males compared to females [28-30]. All subjects underwent structured psychiatric interviews (M.I.N.I.) [31] performed by an experienced psychiatrist and no active Axis I disorders were present. Depression was further assessed using the Beck Depression Inventory [32] with an exclusionary cut-off 5. All participants are right handed and no head injury with unconsciousness during their lifetime.

### Task and procedure

**Figure 1:**
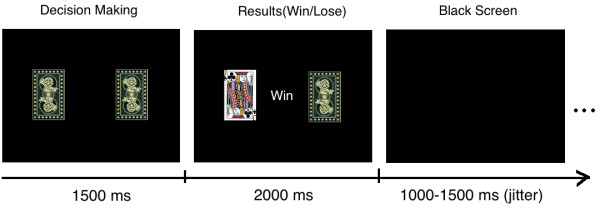
**The timeline of one trial in present task.** First, the backsides of two playing cards were shown and participants were asked to choose either the right or the left card with a button press. After 1.5 s the selected card was turned over and displayed for another 2 s. Depending on the color of the card the participant either won (red playing cards, including the heart and diamond J, Q, K) or lost (black playing cards; including the spade and club J, Q, K) 10 Yuan. After a total presentation time of 2 s, a black screen was presented for 1500 ms.

A reality-simulated guessing task was designed to create gain or loss circumstances. In the task each trial started with the presentation of the backside of two playing cards. Participants were asked to choose either the right or the left card with a button press in 1.5 second. After 1.5 s the selected card was turned over for 2 s. Depending on the color of the card the participant either won (red) or lost (black) 10 Yuan (about 1.6 USD). At the end of this presentation a black screen appeared for 1–1.5 s [33] (Figure 1). The whole task consisted of 245 trials grouped into two blocks, one for 120 trials while the other for 125 trials (only the results of the last 5 trials were totally random, which is to make the final results seems randomly) with one minute between blocks. The whole task runs 1260 seconds (21 minutes). E-prime software (Psychology Software Tools, Inc.) was used to present the task and acquire behavioral data.

At the beginning of each study session, each participant started with a balance of 50 Yuan, and was explicitly informed that he would receive the entire balance in cash at the end of the scanning session. If participant missed to press the key during the selection period, the results will be ‘lose’. Data from any participants choosing the same card for more than 75 percent of all trials or for more than 10 continuous trials were excluded from further analysis (although none of the participants were excluded in this study). This procedure enabled us to control the sequence of wins and losses, and yet gave the participant the impression of free choice.

**Figure 2:**
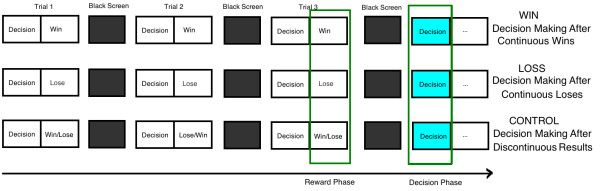
**Three types of conditions used in this study.** We paid attention to two phases during this task: the decision phase and the reward phase. Each phase were observed in three conditions: WIN, decision-making after winning for 3 continuous times; LOSS, decision-making after losing for 3 continuous times; and CONTROL, no repeated wins or losses in the last 3 trials.

Although participants were informed that the outcome of each trial dependent on their choice, the task used a pseudo-random design with three different sequences of trials: (1) Trials after 3 consecutive winning outcomes (WIN). (2) Trials after 3 consecutive losing outcomes (LOSS). (3) Trials after pseudo-random outcomes without more than two consecutive wins or losses (CONTROL). Besides the decision making phase, we also paid attention to the brain activations in reward phase, which can make our results more persuasive (Figure 2).

To increase the number of WIN and LOSS trials within 245 trials for minimizing the scanning time, some trial sequences consisted of four or five continuous win/lose outcomes, and the fourth and fifth trials in these sequences were included in analysis.

### Image acquisition and pre-processing

Imaging data were acquired using a 3 T scanner (Siemens Trio). Structural images covering the whole brain were collected using a T1-weighted three-dimensional spoiled gradient-recalled sequence (176 slices, TR = 1700 ms, TE = 3.93 ms, slice thickness = 1.0 mm, skip = 0 mm, flip angle = 15°, inversion time 1100 ms, field of view = 240*240 mm, in-plane resolution = 256*256). Functional data were acquired using a gradient-echo EPI T2 sensitive pulse sequence (interleaved sequence, 33 slices, 3 mm thickness, TR = 2000 ms, flip angle 90°, field of view 220 × 220 mm^2^, matrix 64 × 64). Stimuli were presented using Invivo synchronous system (Invivo Company, http://www.invivocorp.com/) through a mirror in the head coil.

### Imaging analysis

Imaging was analyzed using SPM5 (http://www.fil.ion.ucl.ac.uk/spm). Preprocessing included slice-timing, motion correction, realignment, normalizing to Montreal Neurological Institute (MNI) space, and spatially smoothing using a 6 mm FWHM Gaussian kernel. We used event related design to analyze the imaging data. SPM5 uses general linear model (GLM) to assess task-related changes in blood oxygen level dependence (BOLD) signal.

Six head-movement parameters derived from the realignment were included as covariates. The design matrix modeled WIN, LOSS, and CONTROL trials in decision making phase and reward phase, separately. In addition to this, we divided WIN and LOSS trials in decision-making phase into WIN-stick, WIN-switch, LOSS-stick, and LOSS-switch conditions according to whether they switched their selection to previous trial. The task set 40 trials for every trial type (WIN, LOSS, CONTROL). However, participants might miss some trials during their selection, and the missed trials were treated as LOSS in our study. Thus, the number of WIN trial was usually less than 40 (33–40).

### Second-level analysis

Second level analysis treated inter-subject variability as a random effect. The images for contrast (WIN-CONTROL, LOSS-CONTROL, WIN-LOSS) of each participant in different phases were imputed into second level one-sample t-test separately. We first identified clusters of contiguously significant voxels at an uncorrected threshold *p* < 0.001, as also used for display purposes in the figures. We then tested these clusters for cluster-level FWE correction *p* < 0.05 and the AlphaSim estimation indicated that clusters with 25 contiguous voxels would achieve an effective FWE threshold p < 0.001 (We actually used 30 contiguous voxels as minimum cluster size). The smoothing kernel used during simulating false-positive (noise) maps using AlphaSim was 6.0 mm, and was estimated from the residual fields of the contrast maps being entered into the one-sample t-test. The formula used to compute the smoothness is that used in FSL (see http://www.fmrib.ox.ac.uk/analysis/techrep/tr00df1/tr00df1/node6.html for more information).

We assessed correlations between changes in BOLD signal in selected region of interest and the behavioral performances to verify our hypothesis. The first correlation was between the changes in BOLD signal (peak beta value) in the inferior frontal gyrus/ACC and differences in reaction times (RTs) between LOSS and CONTROL conditions. The second correlation was between the changes in BOLD signal in the caudate significant clusters (mean value of the three survived clusters) and the number of repeat card choices during WIN. The values of each ROI were extracted using pipeline software Neuroelf (a pipeline fMRI data processing software based on SPM5. Please visit neuroelf.net for more information).

## Results

### Behavioral performance

A repeated-measures ANOVA indicated a significant main effect of trial type WIN (366.98 ± 17.77 ms), LOSS (407.10 ± 21.41 ms), and CONTROL (370.75 ± 17.81 ms)) on response time (RT) [*F*(2,30) = 7.126, *p* = 0.002]. Post-Hoc analysis (LSD) revealed slower RTs in the LOSS relative to WIN or CONTROL trials. RT did not significantly differ between WIN and CONTROL trials (Table 1).

**Table 1:** Comparisons among different conditions in the decision-making task

**Comparisons**	**Mean difference**	**Std. error**	**Sig.**
Response time (ms)			
LOSS-WIN	40.12	13.56	.006
LOSS-CONTROL	36.36	10.98	.002
WIN-CONTROL	−3.77	10.45	.721
Repeat rates			
LOSS-WIN	−1.07	2.19	.629
LOSS-CONTROL	13.93	1.76	.000
WIN-CONTROL	15.00	2.19	.000

To assess whether subjects showed a selection bias in the continuous conditions, we analyzed the repeated choice rates in each condition. Participants tended to repeat the previous choice during both WIN (67.21% ± 9.96) and LOSS (66.14% ± 6.97) conditions, but not in the CONTROL (52.20% ± 6.92) (Table 1).

One important issue in binary choice task is ‘who cares’ thoughts during decision-making, because the outcomes of the choice are independent, random, and equally likely. Previous studies tried to avoid this situation by trying some specific methods. For example, one study tried to avoid this issue by using trinary choice with non-equiprobable events [34]. In this study, the behavioral results (RTs, repeat rates) of different trial types suggested that participants paid attention to the decision process, and did not made choices randomly.

### Brain activations in reward/punishment phase

**Figure 3:**
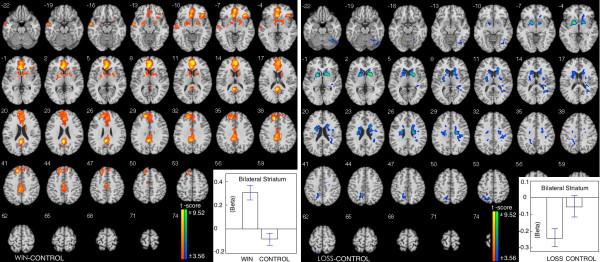
**Brain activations in WIN/LOSS compare to CONTROL in reward phase.** Left, comparison between WIN and CONTROL; Right, comparison between LOSS and CONTROL.

We compared the brain activations in the reward/punishment phase in different conditions. The comparison between WIN and CONTROL showed that higher brain activation was found in WIN trials in bilateral striatum, right ACC and left posterior cingulate cortex. The beta figure showed that the difference in striatum (mean peak value in bilateral striatum) is caused by enhanced brain activation in WIN (Table 2, Figure 3).

**Table 2:** Regional brain activity changes in different comparisons in decision-making and reward phases

**x,y,z**^**a**^	**Peak intensity (t-value)**	**Cluster size**^**b**^	**Region**^**c**^	**Brodmann’s area**
Decision phase				
WIN - CONTROL (Higher activated)				
12, 9, 3	5.93	124	R Ventral Striatum	
−12, 3, 0	5.68	77	L Ventral Striatum	
−12, 48, 30	6.02	210	L Superior Frontal Gyrus	9
LOSS - CONTROL (Higher activated)				
21, 48, 33	6.97	1139	R Superior Frontal Gyrus	9,10
−42,15,-12	7.27	105	L Inferior Frontal Gyrus	47
36, 15, −18	6.94	180	R Inferior Frontal Gyrus	44
3, −21, 33	5.32	33	R Anterior Cingulate Gyrus	23
12, −12, 9	4.96	45	R Thalamus	
−21, −39, 12	5.28	38	L Ventral Striatum	
30, 27, 39	5.78	73	R Middle Temporal Gyrus	8
WIN - LOSS (Higher activated)				
24, −36, 15	5.96	90	R Caudate	
−21, −39, 12	5.63	49	L Caudate	
WIN - LOSS (Lower activated)				
18, 66, 18	−6.57	237	R Superior Frontal Gyrus	10
48, 12, −12	−5.76	149	R Inferior Frontal Gyrus	45
−24, 12, −21	−5.38	31	L Inferior Frontal Gyrus	47
Reward phase				
WIN - CONTROL (Higher activated)				
3, 48, 12	9.21	1929	R Anterior Cingulate	32
−3,-48,27	9.52	638	L Posterior Cingulate	23
12, 6, −3	4.53	157	R Striatum	
−12, 9, −3	4.21	179	L Striatum	
LOSS - CONTROL (Lower activated)				
21, 6, 3	−9.48	689	R Striatum	
−18, 3, −3	−9.50	450	L Striatum	
−15,-54,39	−5.24	125	L Precuneus	7

^a^Peak MNI Coordinates.

^b^We first identified clusters of contiguously significant voxels at an uncorrected threshold p < 0.001, as also used for display purposes in the figures. We then tested these clusters for cluster-level FWE correction p < .05 and clusters with at least 30 voxels were survived. Voxel size = 3*3*3.

^c^The brain regions were referenced to the software Xjview (http://www.alivelearn.net/xjview8) and double checked with atlas.

Lower bilateral striatum activation and left precuneus activation were found in LOSS than in CONTROL. The beta figure showed that the difference in striatum (mean peak value in bilateral striatum) is caused by decreased brain activation in LOSS (Table 2, Figure 3).

### Brain activations in decision-making phase

#### WIN vs. CONTROL

**Figure 4:**
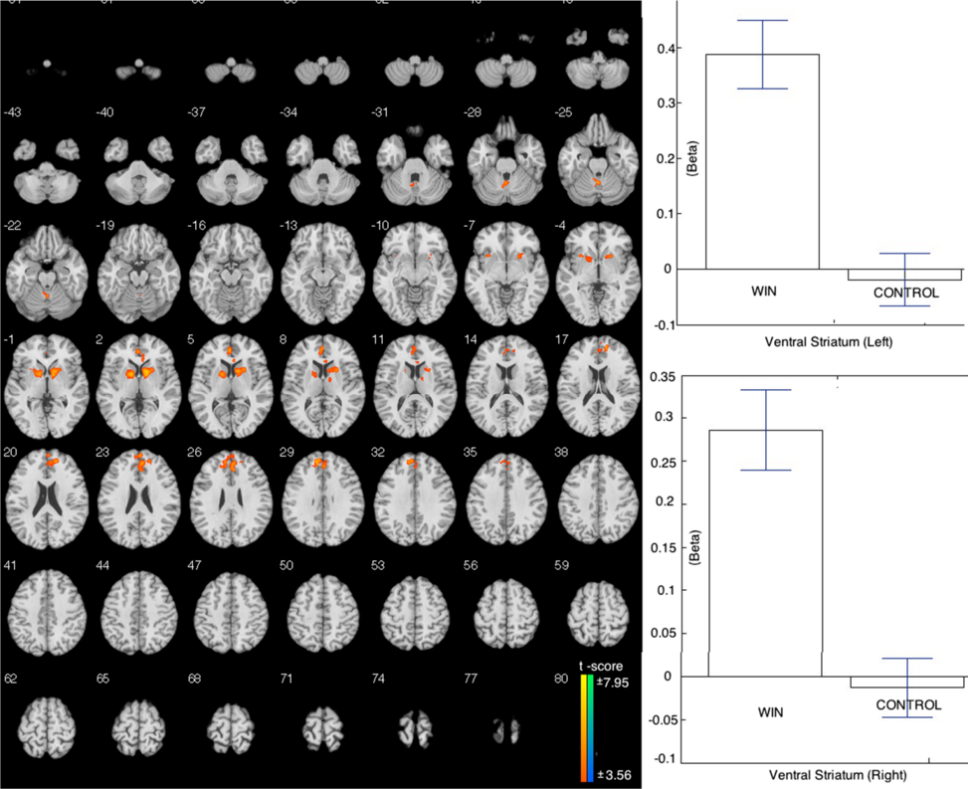
**Brain areas showing stronger activation when comparing WIN to CONTROL.** Left: The activation include bilateral ventral striatum and left superior frontal gyrus. Right: The Beta figures of ventral striatums showed that the difference was driven by the WIN.

During the WIN relative to CONTROL condition, greater BOLD signal was observed in the mesolimbic-frontal regions, including the bilateral ventral striatum and the superior frontal gyrus. The beta figures in bilateral striatum showed that the difference was caused by the enhanced brain activation in WIN (Table 2, Figure 4).

#### LOSS vs. CONTROL

**Figure 5:**
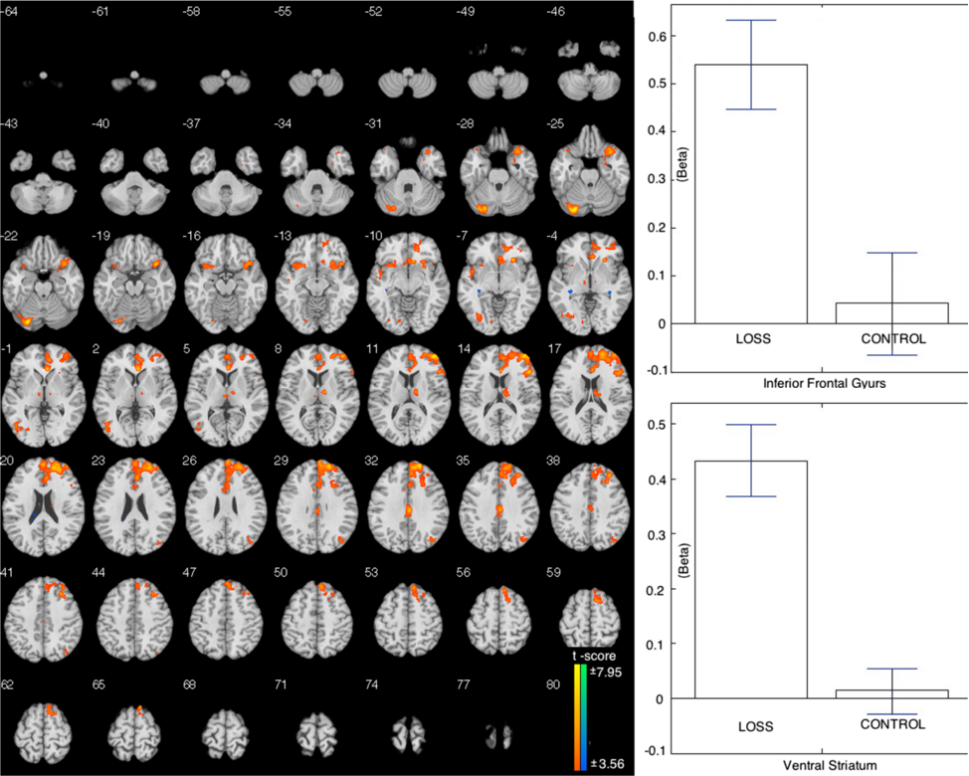
**Brain areas showing stronger activation when comparing LOSS to CONTROL.** Left: The activation including left ventral striatum, anterior cingulate cortex, the thalamus, and middle temporal gyrus. Right: Beta figures showed the difference was driven by LOSS.

The LOSS, relative to CONTROL, showed increased BOLD signal in mesolimbic-frontal regions, including the left ventral striatum and ACC. In addition, the increased signals were also found in the bilateral inferior frontal gyrus, middle temporal gyrus, and thalamus during this process. The beta figures in IFG and striatum showed that the difference is caused by the enhanced brain activation in LOSS (Table 2, Figure 5).

#### WIN vs. LOSS

**Figure 6:**
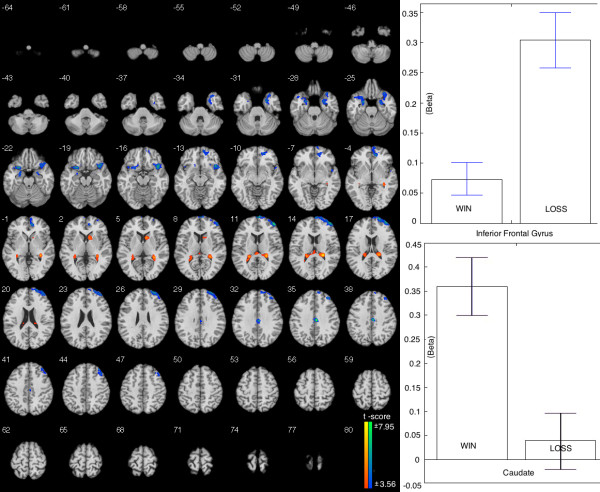
**Brain areas showing different activations when comparing WIN to LOSS.** Left: The increased activation showed in bilateral caudate. Lower activation was found in inferior frontal gyrus and superior frontal gyrus. Right: The Beta figures showed that the difference in IFG was driven by the higher activation in LOSS. However, the difference in caudate was driven by the higher activation in WIN.

The WIN condition showed greater BOLD signal increases in bilateral caudate compared to LOSS. In contrast, the LOSS condition showed greater BOLD signal in the inferior frontal gyrus and superior frontal gyrus compared to WIN (Table 2, Figure 6). The beta figures showed that the difference in IFG is caused by enhance brain activation in LOSS, and the difference I caudate is caused by the enhanced brain activation in WIN.

### Correlation results

**Figure 7:**
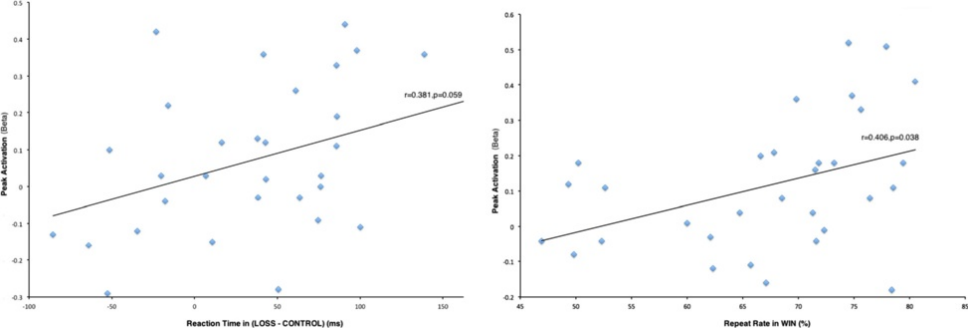
**Correlation between behavioral performance and brain activations in relevant brain regions.** Left: Correlation between mean RTs in LOSS-CONTROL and the brain activity in inferior frontal gyrus in LOSS-CONTROL. Right: Correlation between repeat rates in WIN and the brain activities in caudate in WIN.

First, beta values of the contrast of LOSS versus CONTROL in the inferior frontal gyrus and RT differences between LOSS and CONTROL conditions showed a marginally significant correlation (*r* = 0.381, *p* = 0.059). Second, the beta values of the contrast of WIN versus CONTROL in the caudate clusters (mean value of the two survived clusters) and the rates of repeat card choices during WIN showed a significant correlation (*r* = 0.406, *p* = 0.038) (Figure 7). There is no significant correlation (even marginally significant) between ACC activation and behavioral performance; thus, we did not present the figure here.

### Stick or switch in WIN and LOSS

**Figure 8:**
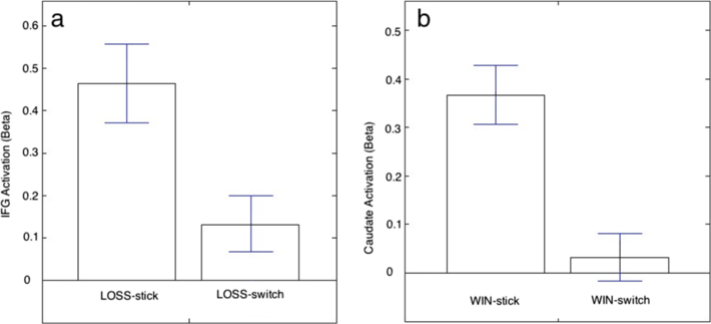
**Brain activities in interested brain regions in stick or switch trials in WIN and LOSS. a**: IFG activation in LOSS-stick and LOSS-switch; **b**: Caudate activation in WIN-stick and WIN-switch.

We grouped WIN and LOSS trials based on whether participants switched choice relative to the previous one. They showed a mean of 27 WIN-stick, 13 WIN-switch, 23 LOSS-stick, and 12 LOSS-switch trials. We paid attention to the activation of related brain areas (Caudate in WIN and IFG in LOSS). The data in further analyses were extracted from the peak values in these ROIs, no matter whether they show differences among different conditions or not. The IFG showed a greater increase in BOLD signal in LOSS-stick than in LOSS-switch (*t* = 4.21, *p* = 0.041) (Figure 8a). The caudate showed a greater increase in WIN-stick relative to WIN-switch (t = 6.35, p = 0.009) (Figure 8b).

## Discussion

Using a task that simulates real-life gambling, we assessed behavioral bias and its neural correlates using fMRI in both WIN and LOSS conditions. In reward phase, the higher striatum activations in WIN compare to CONTROL and the decreased striatum activation in LOSS are consistent with previous findings about the role of striatum in rewarding and punishment (see Reviews [35,36]), these results make the results in decision-making phase more persuasive.

### Enhanced desire to win after WIN and LOSS

Consistent with our hypothesis, mesolimbic regions (ventral striatum, superior frontal gyrus) showed greater activations during WIN and LOSS relative to CONTROL. These findings suggest that human decision-making is modulated by reinforcement learning mechanisms supported by the ventral and dorsal striatum, even when participants are aware that trial-to-trial outcomes are independent and random. Both WIN and LOSS conditions involved stronger activation in the reward system (ventral striatum), which has been associated with stronger desires to win [37]. Previous researches have shown that ventral striatum is associated with reward-related behaviors (obtain reward or avoid punishment) [38,39]. Neuroimaging studies have demonstrated that the ventral striatum responds to a variety of rewarding stimuli, including both social and non-social rewards [40]. In addition, the striatum has also been implicated in choice-outcome contingency learning via feedback, especially when they process the prediction results leading to changes in decision-making [41,42].

According to the features of the task and the activation in the ventral striatum, we speculate that, relative to CONTROL, WIN and LOSS conditions elicit a greater desire to win in the next bet. These findings are consistent with anecdotal reports in pathological gamblers and healthy individuals alike [43] that participants’ desire to win is activated in continuous win situations because they experience it as a streak of good luck; and in continuous loss situations, individuals often want to win back their losses in their next gamble. These cognitive biases may contribute to vulnerability to pathological gambling.

### Gambler’s fallacy after continuous losses

In LOSS, people tend to repeat their previous choices (66.14%). This pattern is known as the gambler’s fallacy. They believe that, after losing so many times, same selection of next bet should generate opposite outcomes (win). The slower RT in LOSS relative to WIN or CONTROL might indicate that participants experienced more conflicts during LOSS trials. Our imaging data showed that decisions in LOSS were associated with greater activities in the prefrontal network when compare WIN to CONTROL. Previous imaging studies have shown that frontal network is involved in cognitive control mechanisms that support flexible, goal-directed behaviors, such as conflict resolution, inhibition of prepotent responses [42,44]. In this study, the higher frontal brain activation in LOSS might suggest that participants engaged more cognitive efforts during the decision-making. In addition to this, LOSS-stick showed higher IFG brain activation than LOSS-switch. Which suggest that LOSS-stick (gambler’s fallacy) need more cognitive endeavor to evaluate the situation and make decisions. The LOSS-switch is an easy way in decision-making; it doesn’t need too much cognitive endeavors.

Functional imaging and lesion studies suggest that affect and emotion play critical roles in decision-making [45]. Continuous losses elicited strong frustration and negative mood, which may disturb the decision-making process and require participants to engage more cognitive effort. In summary, the performance and neural activation in LOSS trials is consistent with gambler’s fallacy, and indicates frustration and greater engagement of executive control as evidenced by enhanced activity in the frontal regions.

### ‘Hot hand’ fallacy after continuous wins

In WIN condition, people also tend to repeat their previous selections (67.21% of the same key). This should not be viewed as gambler’s fallacy although it is similar to LOSS. The RT in WIN is significantly shorter than that in LOSS, which means that subjects just followed their previous win selections without much consideration in WIN than in LOSS. This suggests that less conflicting process or cognitive endeavor was engaged in the decision making process after continuous wins. This is consistent with our imaging data where frontal network showed decreased activities when comparing WIN to LOSS.

Increased activities in caudate were observed when comparing WIN trials to LOSS trials. Significant correlations between the activation in caudate and the repeat rates in WIN condition also support this conclusion. The caudate nucleus was reported to be involved in anticipation and performance-related feedback [26,27]. More interestingly, a recent study using a guessing task with monetary outcomes showed that the caudate was recruited only at the time when participants believed that there were contingencies between their actions and the subsequent results (received a reward or punishment) [46]. The comparison results showed that the caudate activation in WIN-stick is significant higher than in WIN-switch, which means that the process of stick to previous choices involved more caudate activities in WIN. These results provide support to the role of caudate in hot-hand fallacy. Thus, we can speculate that the continuous win reinforced their decisions and resulted in faster and biased responses to the previously rewarded response (the ‘Hot hand’ fallacy).

### Limitations

Although studies have shown that men traditionally have been more likely than women to gamble. However, there is gender difference in the types of gambling (males: strategic-typed preferences in gambling; and females’ gambling preferences: non-strategic forms (e.g. slot machine) [47]). In addition, men were stated as ‘more action-oriented’ while women were described as ‘escape-oriented’ in their motivations of gambling [48]. Thus, only males were included in this study might limit the external validity of current results.

## Conclusions

Human decision-making is often affected by prior selections and their outcomes. In this study, we measured the behavioral performances and brain activations in decision process by creating continuous wins and loss situations. The result is valuable in understanding the behavioral and biological underpinnings during decision making and gambling process.

In conclusion, our study has shown both continuous wins and losses exert influence on following decision-making. First, both in WIN and LOSS, a higher desire to win was elicited (increased activation in the mesolimbic pathway). Second, in the context of continuous losses, the decision making process may require more cognitive control to resolve the conflict/negative emotion (higher frontal brain activation). Gambler’s fallacy may underlie their persistence with a previously punished decision. Third, after winning a gamble, the positive results heightened their confidence in decision-making (increased activities in caudate in WIN), which is known as the ‘hot hand’ fallacy.

## Abbreviations

fMRI: Functional magnetic resonance imaging; RT: Response time; BOLD: Blood oxygen level dependence; FWE: Family-wise-error; GLM: General linear model; MNI: Monteal Neurological Institute; WIN: Decision making after 3 consecutive winning trials; LOSS: Decision making after 3 consecutive losing trials; CONTROL: Control condition; ACC: Anterior cingulate cortex.

## Competing interests

The authors declare that they have no competing interests.

## Authors’ contributions

GD conceived and designed the experiments; GD, XD performed the experiments: XL, HZ analyzed the data, contributed reagents/materials/analysis tools; GD, XL wrote the paper. All authors read and approved the final manuscript.
